# 2202. Influenza Surveillance of Families in an Observational Household Study 2019-2021

**DOI:** 10.1093/ofid/ofac492.1821

**Published:** 2022-12-15

**Authors:** Tara M Babu, Amanda M Casto, Jessica Heimonen, Yongzhe Wang Wang, Annie Emanuels, Eric J Chow, Samara Hoag, James Hughes, Constance E Ogokeh, Melissa A Rolfes, Timothy M Uyeki, Lea Starita, Janet A Englund, Helen Y Chu

**Affiliations:** University of Washington, Seattle, Washington; University of Washington, Seattle, Washington; University of Washington, Seattle, Washington; University of Washington, Seattle, Washington; University of Washington, Seattle, Washington; Public Health - Seattle & King County, Seattle, Washington; Seattle Public Schools, Seattle, Washington; University of Washington, Seattle, Washington; Centers for Disease Control and Prevention, Atlanta, Georgia; Centers for Disease Control and Prevention, Atlanta, Georgia; Centers for Disease Control and Prevention, Atlanta, Georgia; University of Washington, Seattle, Washington; Seattle Children's Hospital/ Univ. Washington, Seattle, Washington; University of Washington, Seattle, Washington

## Abstract

**Background:**

Families with children may be at higher risk for influenza infection. Community transmission can suffer from underreporting as testing is often not performed. We studied the epidemiology of influenza in households with school-aged children using home-based sample collection.

**Methods:**

We conducted a remote household study surveilling respiratory viruses from November 2019-June 2021, in King County, Washington (WA), USA. Households with school-aged children were enrolled, mailed home specimen collection kits, and asked to self-assess for weekly acute respiratory illness (ARI) using remote survey platforms. Participants with ARI symptoms were prompted to complete serial illness surveys and self-collect/parent collect mid-turbinate nasal swabs. Samples were sent to a University of Washington study laboratory for RT-PCR influenza testing. Influenza rates were compared to WA Department of Health (DOH) reporting.

**Results:**

A total of 1861 ARI events were reported among 992 adults and 869 children in 470 households; 75 influenza cases were detected (36 influenza A and 39 influenza B). The study participant median age was 32 years (0-84), 10 years (1-49) for influenza A, and 11 years (3-49) for influenza B cases. Overall 13% of households had an influenza case, of which 13 (22%) reported >1 case. A total of 81% of participants reported receipt of one dose of the 2019-2020 influenza vaccine, including 91% of influenza A and 90% of influenza B cases, and 84% received the 2020-2021 influenza vaccine. Like WA DOH, we observed a wave of influenza B cases followed by influenza A in 2019-2020. During influenza season 2020-2021, WA DOH reported 9 positive influenza tests and none observed in our study. Commonly, influenza case-patients reported were fever, cough, rhinorrhea, and fatigue. GI symptoms were more common in children than adults. Of the cases, 92% of influenza A and 78% of influenza B occurred in children.

**Figure 1.**
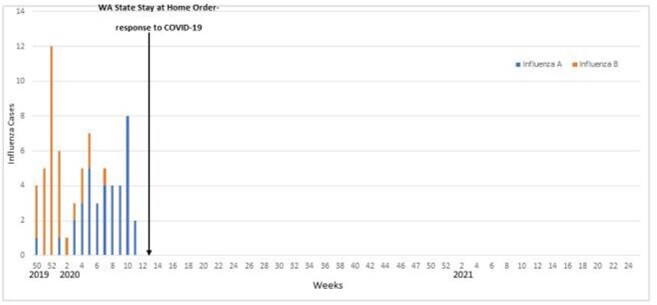
Influenza A and B cases from 2019-2021

**Figure 2A.**
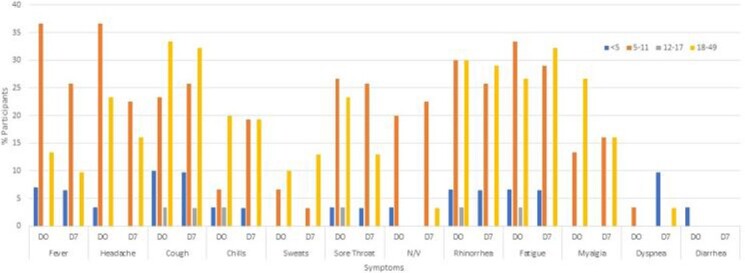
Reported Symptoms of Influenza A

D0-Day of reported onset, D7-7 days after reported illness onset. No participants >49 years were positive for influenza. D0: 30 participants responded and of respondents, 13% <5 years, 47% 5-12 years, 3% 13-17 years, and 37% 18-49 years. D7: 31 participants responded and of respondents 13% <5 years, 48% 5-11 years, 3% 12-17 years, and 36% 18-49 years.

**Figure 2B.**
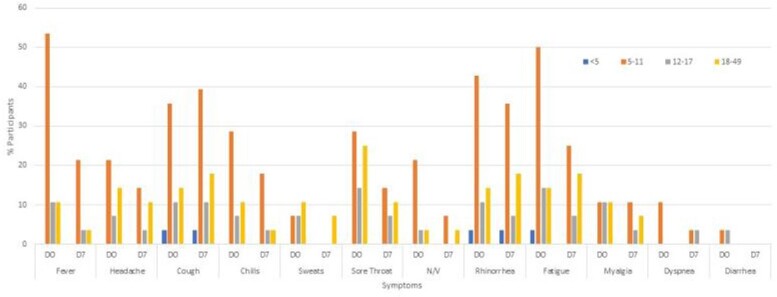
Reported Symptoms of Influenza B

D0-Day of reported onset, D7-7 days after reported illness onset. No participants >49 years were positive for influenza. D0: 28 participants responded and of respondents, 4% <5 years, 57% 5-12 years, 14% 13-17 years, and 25% 18-49 years. D7: 28 participants responded and of respondents, 4% <5 years, 57% 5-11 years, 18% 12-17 years, and 21% 18-49 years.

**Conclusion:**

Influenza illness in 2019-2020 was initially influenza B, and subsequently replaced by influenza A. Most cases were in children and adolescents, despite at least one dose of influenza vaccine. Symptoms were widely distributed and similar between influenza A and B. Influenza incidence in our cohort declined to zero with the rise of SARS-CoV-2 cases and widespread mitigation efforts.

**Disclosures:**

**Janet A. Englund, MD**, AstraZeneca: Advisor/Consultant|AstraZeneca: Grant/Research Support|GlaxoSmithKline: Grant/Research Support|Meissa Vaccines: Advisor/Consultant|Merck: Grant/Research Support|Pfizer: Grant/Research Support|Sanofi Pasteur: Advisor/Consultant **Helen Y. Chu, MD, MPH**, Cepheid: Reagents|Ellume: Advisor/Consultant|Gates Ventures: Grant/Research Support|Merck: Advisor/Consultant|Pfizer: Advisor/Consultant.

